# A Polymorphism Within the *MBP* Gene Is Associated With a Higher Relapse Number in Male Patients of Multiple Sclerosis

**DOI:** 10.3389/fimmu.2020.00771

**Published:** 2020-05-05

**Authors:** Laura Espino-Paisán, Teresa Agudo-Jiménez, Isabel Rosales-Martínez, Pilar López-Cotarelo, María Ángel García-Martínez, María Inmaculada Domínguez-Mozo, Silvia Pérez-Pérez, Romina Dieli-Crimi, Manuel Comabella, Elena Urcelay, Roberto Álvarez-Lafuente

**Affiliations:** ^1^Laboratorio de Investigación en Genética de Enfermedades Complejas, Instituto de Investigación Sanitaria del Hospital Clínico San Carlos (IdISSC), Red Española de Esclerosis Múltiple (REEM), Madrid, Spain; ^2^Grupo de Investigación de Factores Ambientales en Enfermedades Degenerativas, Instituto de Investigación Sanitaria del Hospital Clínico San Carlos (IdISSC), Red Española de Esclerosis Múltiple (REEM), Madrid, Spain; ^3^Servicio de Inmunología, Hospital Universitari Vall d’Hebron (HUVH), Diagnostic Immunology, Vall d’Hebron Research Institute (VHIR), Barcelona, Spain; ^4^Servei de Neurologia-Neuroimmunologia, Centre d’Esclerosi Múltiple de Catalunya (Cemcat), Institut de Recerca Vall d’Hebron (VHIR), Hospital Universitari Vall d’Hebron, Universitat Autònoma de Barcelona, Red Española de Esclerosis Múltiple (REEM), Barcelona, Spain

**Keywords:** multiple sclerosis, SNP, HHV-6, myelin basic protein, relapse, sex differences, genetic marker

## Abstract

Myelin basic protein (MBP) is thought to be one of the key autoantigens in multiple sclerosis (MS) development. A recent study described the association of the single nucleotide polymorphism (SNP) rs12959006, within the *MBP* gene, with a higher risk of relapse and worse prognosis. We aim at studying potential associations of this SNP to MS in an independent population. Clinical data of the first 5 years of the disease were collected retrospectively from 291 MS confirmed patients. *MBP* polymorphism rs12959006 was genotyped in all patients. Associations with EDSS, number of relapses and serology for Herpesvirus 6 (HHV-6) and Epstein Barr (EBV) viruses were studied. Lymphocyte activation measured by CD69 expression was also analyzed according to sex and rs12959006 genotype. The rs12959006 polymorphism contributed significantly to a higher number of relapses at 5 years after onset only in male patients (rs12959006^∗^TT β = 0.74 [0.36–1.09]; *p* = 7 × 10^–5^). Titers of anti-HHV6 IgG antibodies showed also a mild association with relapses, both in male and female patients (β = 0.01 [0.01–0.02]; *p* = 3.7 × 10^–8^). Both the genetic variation in *MBP* and HHV-6 infection aid in predicting a higher number of relapses during the first years of MS. The association described in *MBP* rs12959006^∗^T is exclusive to male patients.

## Introduction

Multiple sclerosis (MS) is a chronic inflammatory and demyelinating disease affecting the central nervous system (CNS) and is one of the primary neurological causes of physical disability in young adults (1). The underlying mechanism seems to be an autoimmune response to unidentified antigens in the CNS. One of these proposed antigens is the myelin basic protein (MBP), the second most abundant component of the myelin sheath, and a protein that plays an important role in the myelination process. Interestingly, MBP is part of the peptide cocktail used for inducing experimental autoimmune encephalomyelitis (EAE) in mice, along with proteolipid protein (PLP) and myelin olygodendrocyte glycoprotein (MOG) (2). Although MBP has not been proved to be the key autoantigen in MS, MBP-specific autoreactive T-cells have been found in blood of MS patients at a higher rate than in healthy individuals (3).

Several studies have focused on finding new markers for MS diagnosis and prognosis that are cost-effective, reliable, easy to implement and as minimally invasive as possible. Many candidates have been singled out in individual studies (4), but a necessity exists to replicate these findings in independent cohorts to ascertain the validity and reproducibility of these new potential biomarkers. Genetic markers are especially interesting due to their easy determination, stability through time and accessibility of the sample. Genome-wide association studies (GWAS) have identified more than 200 polymorphisms associated with susceptibility to MS (5). Additional studies with carefully selected patients are warranted to find genetic markers that can be used as predictive and prognostic biomarkers for MS.

A recent study pointed to polymorphism rs12959006 within the *MBP* gene as a risk modifier of conversion to definite MS and progression of the disease (6). The authors studied genetic markers in a prospective cohort after a first demyelinating event. They found that the rs12959006 minor allele T was associated with a higher risk of conversion to MS, and a greater hazard of relapse (HR = 1.74 [1.19–2.56]; *p* = 0.005) and annualized disability progression (β = 0.18 [0.06–0.30]; *p* = 0.004). Moreover, in this study, the risk genotype carrying the T allele showed an interaction with baseline titers of IgG antibodies against Human Herpesvirus 6 (HHV-6). We aimed at studying this polymorphism and its putative interaction with viral antibodies against viruses relevant to MS in an independent cohort of MS-confirmed patients.

## Materials and Methods

### Patients

A total of 291 patients (66.7% female) were included in the study. Patients were diagnosed with relapsing remitting multiple sclerosis (RRMS) between 1991 and 2014 according to the Poser or McDonald criteria (7–10), and followed in the Multiple Sclerosis Unit at Hospital Clinico San Carlos (Madrid, Spain). Mean age at onset was 29.40 +7.78 years. Medical records of each patient were reviewed and data from the first 5 years after a first demyelinating event were extracted. Patients were selected based on two criteria: the patient was followed in the unit from MS onset and clinical data such as relapse and EDSS for the first 5 years of the disease were available. Due to a slightly higher availability of relapse data, this variable was chosen as inclusion criterion over EDSS. Written informed consent was obtained from each patient and the study was approved by the Ethics Committee of Hospital Clínico San Carlos.

Clinical data collected included EDSS at 2 and 5 years after onset, relapse in the 30 days prior to EDSS measurement, number of relapses at 2 and 5 years after onset, age at disease onset and disease modifying treatments employed during the evaluated period. Titer of anti-HHV-6 (IgG and IgM) and anti-EBV (EBNA-1 and VCA) antibodies, against two viral agents related to MS that have been previously associated to disease course, were also determined.

Seventy-one percent of patients received disease modifying treatments during the 5 year follow-up, with 28.3% (82 patients) remaining without treatment; among the treated patients, 85.6% (178) received only first line treatments (interferon-β and/or glatiramer acetate) during the period of study. The remaining 14.4% (30) patients received a combination of first line and second line treatments (natalizumab or mitoxantrone, among others). There were no statistically significant differences in the groups studied concerning the distribution of treatment options (no treatment, first line therapies, combined use of first line and second line therapies). To adjust for treatment effect, all multivariable regression models included two variables: “DMT use,” defined as no treatment versus use of any disease modifying treatment in the first 5 years of the disease, and “Onset before 2002,” to account for the reduced treatment options and different therapy approaches before the availability of natalizumab and other second line treatments. The year 2002 was selected because a Spanish patient with onset of disease in that year would have the option to be treated with natalizumab at least 1 year in the 5 year follow-up considered for the study.

Viral antibody data were available for a maximum of 227 patients. In several cases, viral antibody measurement was performed after the 5-year period considered for evaluation (mean duration of MS until antibody determination was 7.47 ±4.78 years). Statistical tests were performed to compare antibody titers from patients evaluated close to diagnosis and those with a longer evolution of disease and no differences were found. Despite these results, antibody titers were always adjusted by duration of disease until measurement when used in regression studies. Viral antibodies were determined in serum samples with ELISA commercial kits: ELISA-VIDATEST anti-HHV-6 IgG and IgM (Vidia), Captia EBNA-1 IgG and VCA-EBV IgG (Trinity Biotech), according to the manufacturer’s protocols.

### Genotyping

Patients were genotyped for the *MBP* polymorphism rs12959006^∗^C > T with Taqman assay C___3079229_20 in a 7900HT Fast Real-Time PCR system (Life Technologies, United States). The genotyping success rate was 100%.

### *In vitro* Activation Analysis and Flow Cytometry

Peripheral blood mononuclear cells (PBMCs) from 75 patients were selected and divided in 4 groups according to sex and genotype at rs12959006 (approximately 19 patients per group). Due to the low frequency of the minor homozygote, only major homozygote and heterozygote patients were included. Treatment effect was accounted for by including patients with the same treatment in every group. Samples were cultured in RPMI supplemented with 10% FBS, 1% penicillin-streptomycin and 1% L-glutamine (Sigma-Aldrich, Bremen, Germany), without stimulus or treated with 5 μg/ml Concanavalin A (Con A, Sigma-Aldrich, Bremen, Germany). Afterward, they were kept at 37°C and 5% CO_2_ for 24 h. Cells were stained with anti-CD69-FITC, anti-CD3-PE, and 7-AAD to exclude non-viable cells (Biolegend, San Diego, CA, United States). Samples were processed and analyzed in a Cytomics FC500 flow cytometer with Kaluza 2.0 software (Beckman Coulter, Brea, CA, United States) and percentage of CD69^+^ cells and median fluorescence intensity (MFI) of CD69^+^ cells in CD3^+^ and CD3^–^ populations were measured. Final values were calculated by extracting the basal CD69 measures (without stimulus) to the samples treated with Con A.

### Statistical Analysis

EDSS and number of relapses at 5 years after onset were evaluated. Data at 2 years after onset were used to confirm the obtained results at a different time point. For multivariable analysis, all variables were analyzed in a correlation matrix with Pearson and Spearman coefficients. Relevant clinical features that showed significant correlation with the dependent variable were thus selected, and models were built to check for joint contribution and to adjust for potential confounding variables. Influence of rs12959006 in EDSS was analyzed with linear regression; influence in relapses, with Poisson regression. Linear regression analyses were performed with SPSS v15.0.1, Poisson regression analyses with RStudio v1.2.5019, and graphical representations were carried out with GraphPad Prism V5.01.

## Results

All participants were genotyped for rs12959006 (minor allele T frequency = 0.18). Allele frequencies were concordant with those reported for the Iberian population in the 1000 Genomes database.

We aimed at studying the contribution of rs12959006 genotype in relapse and EDSS at 5 years after onset. For relapse, we analyzed the number of relapses by genotype using Poisson regression. The rs12959006^∗^TT genotype showed a statistically significant association with higher number of relapses (β = 0.32 [0.07–0.55]; *p* = 0.008) in all patients. Surprisingly, when stratified by sex, both rs12959006 genotypes carrying the minor allele T showed association only in male patients (Table 1).

**TABLE 1:** Poisson regression to test for influence of rs12959006 genotype in number of relapses at 5 years after MS onset.

	**Variables included**	**β(95%CI)**	***p***
MS overall	rs12959006*CC	Reference	Reference
*N* = 291	rs12959006*CT	0.07 (−0.04 to 0.19)	0.24
	rs12959006*TT	0.32 (0.07–0.55)	0.008
MS males	rs12959006*CC	Reference	Reference
*N* = 97	rs12959006*CT	0.21 (0.00–0.41)	0.03
	rs12959006*TT	0.68 (0.35–0.98)	1.8 × 10^–5^
MS females	rs12959006*CC	Reference	Reference
*N* = 194	rs12959006*CT	−6.55 × 10^–5^ (−0.15 to 0.15)	0.99
	rs12959006*TT	−9.99 × 10^–2^ (−0.53 to 0.28)	0.63

*Coefficients (β) and 95% confidence intervals of the variables included are presented. Major homozygote is set as a reference in each analysis. *N*: number of patients in each group.*

We investigated other variables that correlated significantly with relapse to build a multivariable model. Out of the four viral serology studies (IgG and IgM for HHV-6 virus and EBNA-1 and VCA for EBV) only IgG anti-HHV-6 antibodies showed correlation and were included in further statistical analyses, corrected by duration of disease until antibody measurement. Use of DMT during the 5 years evaluated was also included, and adjusted for “Onset before 2002” (as detailed in the Methods section), to account for treatment effect. When analyses were re-run, the rs12959006^∗^TT genotype showed a slightly stronger statistical association with relapse at 5 years after onset in the general population (β = 0.36 [0.08–0.63]; *p* = 0.009) (Table 2). In patients stratified by sex, both rs12959006^∗^CT and TT genotypes showed statistically significant association with a higher number of relapses only in male patients (Table 2), with a stronger effect of the rs12959006 minor homozygote (β = 0.74 [0.36–1.09]; *p* = 7 × 10^–5^).

**TABLE 2:** Poisson regression models for number of relapses at 5 years after MS onset.

	**Variables included**	**β(95%CI)**	***p***
MS overall	rs12959006*CC	Reference	Reference
*N* = 224	rs12959006*CT	0.08 (−0.06 to 0.21)	0.27
	rs12959006*TT	0.36 (0.08–0.63)	0.009
	Anti-HHV-6 IgG antibodies	0.01 (0.01–0.02)	3.7 × 10^–8^
	MS duration until antibody measurement	0.02 (0.00–0.03)	0.009
	DMT use	0.25 (0.02–0.48)	0.04
	Onset of MS before 2002	0.03 (−0.11 to 0.16)	0.68
Males MS	rs12959006*CC	Reference	Reference
*N* = 77	rs12959006*CT	0.26 (0.03–0.48)	0.02
	rs12959006*TT	0.74 (0.36–1.09)	7 × 10^–5^
	Anti-HHV-6 IgG antibodies	0.01 (0.00–0.02)	0.02
	MS duration until antibody measurement	0.01 (−0.01 to 0.03)	0.44
	DMT use	0.63 (0.20–1.07)	0.004
	Onset of MS before 2002	−0.15 (−0.40 to 0.09)	0.23
Females MS	rs12959006*CC	Reference	Reference
*N* = 147	rs12959006*CT	−0.03 (−0.21 to 0.15)	0.73
	rs12959006*TT	0.06 (−0.43 to 0.49)	0.80
	Anti-HHV-6 IgG antibodies	0.02 (0.01–0.02)	1.4 × 10^–6^
	MS duration until antibody measurement	0.03 (0.01–0.05)	0.01
	DMT use	0.12 (−0.14 to 0.39)	0.37
	Onset of MS before 2002	0.09 (−0.07 to 0.26)	0.29

*MS duration until antibody measurement was included in each model to adjust for time until viral antibody determination. DMT use reflects the use of a disease modifying treatment in the 5 years studied versus no treatment. “Onset before 2002” was included to adjust “DMT use” for the availability of second line treatments. Coefficients (β) and 95% confidence intervals of the variables included are presented. Mayor homozygote is set as reference (with no effect) in each analysis. *N*: number of patients in each group.*

Interestingly, titers of IgG anti-HHV6 antibodies were independently associated with higher number of relapses in both male and female MS patients (Table 2).

We attempted at replicating these observations with relapse data of the first 2 years of the disease. Although the effect of rs12959006 was weaker, when evaluating carriers of rs12959006^∗^T it was observed that the presence of the minor allele in male patients was associated with a higher number of relapses (β = 0.30 [0.02–0.57]; *p* = 0.03) while it showed no effect in female patients (β = −0.05 [−0.12 to 0.80]; *p* = 0.78) or in the general population (β = 0.11 [−0.07 to 0.68]; *p* = 0.23).

For EDSS we built multivariable linear regression models with rs12959006 and the other selected variables and no statistically significant contributions were found.

**FIGURE 1:**
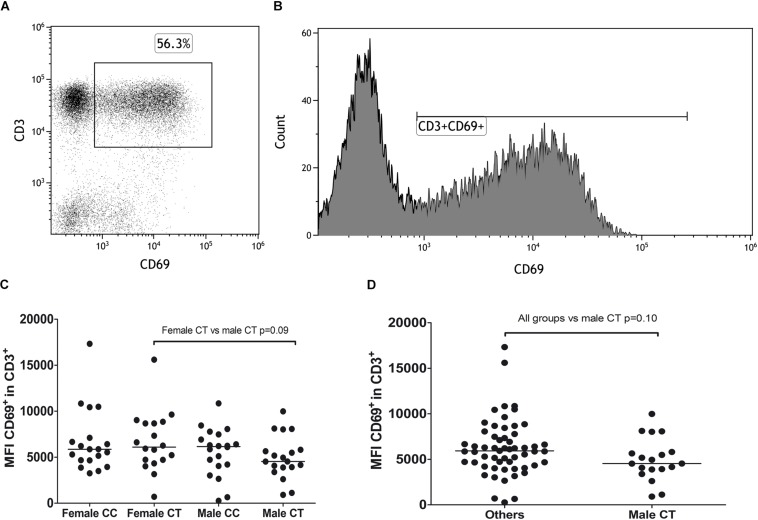
Expression of CD69 in CD3^+^ cells measured as MFI according to sex and genotype at rs12959006. **(A)** Dot plot showing the selection of CD3^+^CD69^+^ lymphocytes. **(B)** Histogram showing the selection of CD3^+^CD69^+^ population for MFI measurement. **(C,D)** Statistical comparison of CD69 expression in CD3^+^ cells, measured as median fluorescence intensity (MFI), between the four groups studied **(C)** and all groups vs. male CT **(D)**. Median of each group is represented.

To evaluate whether potential alterations in immune cells correlated with the presence of the rs12959006^∗^T allele, we analyzed activation in PBMCs of MS patients through stimulation with Con A and study of CD69 expression in CD3^+^ and CD3^–^ cells. Patients were stratified by sex and genotype at rs12959006, and the percentage of positive cells and MFI of CD69 were studied. Due to the low frequency of the rs12959006^∗^TT genotype, only the heterozygote and major homozygote were analyzed. A trend for a lower expression of CD69 in male patients carrying rs12959006^∗^T was observed, although it was not statistically significant (female CT vs. male CT *p* = 0.09; Figure 1).

## Discussion

Our study aims at analyzing the putative associations of the *MBP* polymorphism rs12959006 with MS. A previous study reported associations of this polymorphism with conversion to MS and worse prognosis in these patients (6). The authors studied an Australian cohort of 127 patients with a first demyelinating event compatible with MS. Patients were followed for 5 years. Only 68 patients developed confirmed MS within the duration of the study and they experienced a total of 152 relapses versus the 1292 relapses registered in our study group of 291 MS-confirmed patients. Our analysis benefits from higher statistical power. We describe an association between rs12959006 and risk of a higher number of relapses at 2 and 5 years after MS onset. Interestingly, we observe this association only in male MS patients. MS presents sex differences, being more prevalent in females but more severe in males (11, 12). Recent studies have pointed to other genetic polymorphisms that affect MS only in male patients (13, 14) that could explain part of the difference in prevalence and course of MS between sexes.

We observe a trend for a lower expression of CD69 in activated CD3^+^ cells in male patients carrying rs12959006^∗^T allele. Several studies associate the blocking or lower expression of CD69 in T cells with an exacerbation of autoimmune diseases (15, 16) and a higher induction of pro-inflammatory Th17 cells in mice (17). Moreover, CD69^+^ T regulatory cells have been reported to have higher immunosuppressive potential in the mouse model of colitis (18). A lower expression of CD69 in T cells could be related to a defective control of inflammation and a worse evolution of autoimmune diseases. In the light of previous knowledge, it would be interesting to replicate the observed trend in rs12959006^∗^CT patients and to extend it to the low-frequency rs12959006^∗^TT genotype.

We observe that levels of anti-HHV6 IgG antibodies are positively associated with number of relapses. Other studies (19, 20) have previously described associations between high or increasing anti-HHV-6 antibody titers and risk of relapse, and even pointed to a possible predictive value of anti-HHV-6 IgG and IgM levels (19). An elevated antibody titer could suggest a stronger immunological response to HHV-6 infection. Previous studies compared the molecular structure of MBP and HHV-6 proteins. MBP and peptide U24 of the HHV-6 viral particle share an identical sequence of 6 aminoacids (MBP residues 96–102, HHV-6 U24 residues 4–10), pointing at a possible contribution of viral infections to MS risk through molecular mimicry. A classic study (3) showed that T-cells cross-reactive to both MBP and HHV-6 were more abundant in MS patients than in healthy controls, an observation that was replicated in a more recent study in Chinese population (21). These studies point to the idea that the joint effect of HHV-6 infection and its molecular mimicry with MBP could be important in MS pathogenesis.

For future research, it would be interesting to replicate these observations in a group with baseline anti-HHV-6 IgG antibody measurements and in the context of a prospective study to determine the usefulness of rs12959006 in clinical practice.

Our study, and those from others, consolidates the contribution of rs12959006, within the *MBP* gene, as a marker to predict MS activity. Furthermore, according to our results, there seems to be an influence of both the genetic polymorphism in *MBP* and HHV-6 infection in predicting a higher relapse rate that is exclusive to male MS patients.

## Data Availability Statement

All datasets generated for this study are included in the article/supplementary material.

## Ethics Statement

The studies involving human participants were reviewed and approved by the Comité Ético de Investigación Clínica, Hospital Clínico San Carlos, Madrid (Spain). The patients/participants provided their written informed consent to participate in this study.

## Author Contributions

LE-P, TA-J, IR-M, PL-C, MG-M, MD-M, SP-P, and RD-C performed the experiments and collected the clinical data. LE-P, MC, EU and RÁ-L designed the experiments, analyzed the data, and drafted the manuscript.

## Conflict of Interest

The authors declare that the research was conducted in the absence of any commercial or financial relationships that could be construed as a potential conflict of interest.
